# Displacement Measurement Based on the Missing-Order Talbot Effect

**DOI:** 10.3390/s25010292

**Published:** 2025-01-06

**Authors:** Liuxing Song, Kailun Zhao, Xiaoyong Wang, Jinping He, Guoliang Tian, Shihua Yang, Yaning Li

**Affiliations:** 1 College of Astronautics, Nanjing University of Aeronautics and Astronautics, Nanjing 211106, China; 2 Beijing Institute of Space Mechanics and Electricity, Beijing 100081, China

**Keywords:** Talbot effect, displacement measurement, grating, missing-order Talbot effect, FFT

## Abstract

Displacement measurement is a crucial application, with laser-based methods offering high precision and being well established in commercial settings. However, these methods often come with the drawbacks of significant size and exorbitant costs. We introduce a novel displacement measurement method that utilizes the missing-order Talbot effect. This approach circumvents the need to measure contrast in the Talbot diffraction field, opting instead to leverage the displacement within the missing-order Talbot diffraction pattern. Our method only requires parallel light, an amplitude grating, and a detector to achieve displacement measurement. The measurement dynamic range can be adjusted by altering the grating period and the wavelength of the incident light. Through careful simulation and experimental validation, our method exhibits a correlation coefficient *R* surpassing 0.999 across a 30 mm dynamic range and achieves a precision superior to 3 μm.

## 1. Introduction

Displacement measurement is a critical application in various fields, and while laser-based methods have led the field with their precision, they come with significant drawbacks, such as their large size and high costs. Laser-based displacement detection devices are primarily divided into two main categories: time-of-flight (ToF) laser ranging and laser interferometry. ToF laser ranging calculates distance by timing the flight of a light pulse from emission to reflection, providing the benefits of rapid measurement, making it particularly suitable for long-distance metrology. However, in short-range measurements, ToF methods require extremely high temporal resolution, which can lead to significant errors, limiting their precision to the sub-millimeter level [1]. On the other hand, laser interferometry achieves sub-wavelength precision through the interference of light waves, but this precision comes at the cost of requiring sophisticated equipment, which leads to higher expenses [2].

The Talbot effect, renowned for its unique diffraction pattern and characterized by miniaturization and low cost, has been extensively applied in various fields including angular sensing [3,4], wavefront sensing [5], and photolithography [6,7]. This phenomenon occurs with the illumination of periodic objects by a plane wave, prompting the periodic generation of self-images along the direction of the incident light [8]. Characterized by fluctuations in the contrast of these self-images within the Talbot diffraction field in relation to distance, the effect produces distinct high-contrast fringes at particular intervals, identified as Talbot distances ($Z_{t}$). These distances are determined by both the wavelength (λ) and the grating period (*d*), and are described by the equation $Z_{t}=2n\frac{d^{2}}{\lambda }$ [9].

The unique characteristics of the Talbot diffraction patterns have sparked considerable interest in academia for their potential in displacement measurement. P. Chavel pioneered the application of the Talbot effect for displacement measurement, employing variations in received self-imaging intensity to scan and detect object contours [10]. Building on this foundation, G. Spagnolo advanced the field of displacement measurement by employing a 200 μm period grating, achieving a linearity better than 1% through frequency-domain processing [11]. Furthermore, Francisco expanded the technique’s application to measure the roughness of stepped surfaces with a single grating [12]. Research then focused on dual-grating systems, which convert diffraction patterns into intensity information using two sets of gratings. Luo employed a dual-grating arrangement to determine lens focal lengths [13], while Xin leveraged small-period gratings for precise displacement tracking [14]; these findings underscore the Talbot effect’s aptitude for high-precision displacement quantification. P. Rodriguez-Montero replicated the dual grating using the photo-emf effect, achieving a resolution close to 10 μm over a 1.5 mm dynamic range [9]. In addition to the grating period, wavelength also plays a significant role in influencing the Talbot diffraction field. Vela-Esparza utilized a dual-wavelength Talbot diffraction field to measure displacement [15], while Satish Kumar Dubey improved the precision and expanded the dynamic range through multi-wavelength scanning of stepped surfaces [16]. Furthermore, E. Tepichin-Rodriguez extended the technique to include the measurement of stepped surfaces using spherical waves [17]. Although these studies have successfully measured displacement by relying on the periodic changes within the Talbot diffraction field, they have overlooked the potential value of the ‘missing’ regions beyond the Talbot zones.

When a finite-sized plane light source illuminates a grating, the regions beyond the Talbot self-imaging areas are characterized by the formation of stripe-like images due to the absence of specific diffraction orders, marking a distinct difference from the patterns observed within the Talbot region. E. A. Hiedemann observed these stripe-like images outside the Talbot region [18], and Silva, D. E. termed this phenomenon the “walk-off effect” [19]. John offered an explanation of this effect in the frequency domain, yet the explanation was not entirely intuitive [20]. Rao provided an innovative visual interpretation of the Talbot diffraction field from the perspective of wave optics, but this interpretation did not cover the regions where diffraction orders are missing [21]. There have been scant applications related to this phenomenon. To our knowledge, Ezaki is the pioneer in harnessing the missing-order phenomenon in photolithography for the creation of high-aspect-ratio structures [7]. In this paper, we refer to this phenomenon as the “missing-order Talbot effect” and demonstrate its application in achieving high-precision displacement measurements.

We present a novel approach that harnesses the missing-order Talbot effect for the first time in displacement measurement. This method offers significant advantages over traditional Talbot diffraction regions by providing a larger dynamic range and superior linearity. Our proposed algorithm has been rigorously validated through both simulation and experimental testing, demonstrating a correlation coefficient as high as 0.999 within a 30 mm dynamic range, with the highest precision exceeding 3 μm. The precision of our method can also be further enhanced by reducing the grating period. Leveraging its compact size and cost-effectiveness, this method holds great potential for future applications in optical alignment, robotic arm sensing, and industrial inspection, among other fields.

## 2. Theory

### 2.1. The Principle of the Talbot Effect and Missing-Order Talbot Effect

**Figure 1:**
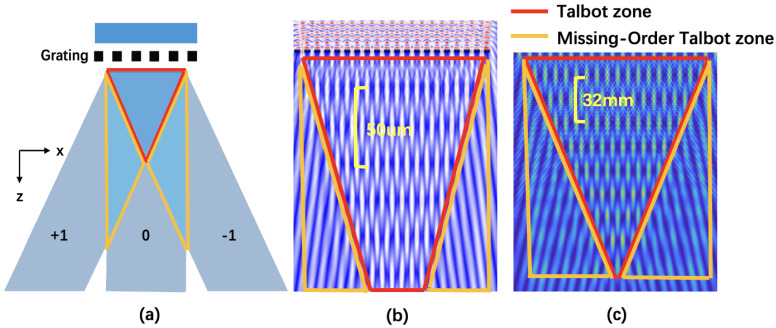
Schematic and simulation of the Talbot effect. (**a**) Illustrates the formation of Talbot zones (red) where the 0th and ±1st diffraction orders overlap, creating periodic self-imaging, and missing-order Talbot zones (orange) that produce stripe-like images due to the absence of certain diffraction orders. (**b**) Shows the diffraction field for a 4 μm grating period under 632 nm illumination, with a Talbot distance of 50 μm, as calculated by FDTD. (**c**) Displays the diffraction field for a 100 μm grating period under the same illumination, with a Talbot distance of 32 mm, calculated using the angular spectrum method.

The illustration of the Talbot effect, as shown in Figure 1a, depicts a plane wave that, after passing through a grating, diffracts into multiple orders of light. Using the 0th and ±1st orders as examples, the areas encompassed by these orders are known as the Talbot regions. These regions are characterized by the periodic generation of the grating’s self-images, which are indicated by the red markings in the figure. As the wave propagates, the orange regions selectively capture only the 0th and +1st, or 0th and −1st diffraction orders, leading to the formation of stripe-like images. These specific areas, where certain diffraction orders are absent, are termed missing-order Talbot regions. This phenomenon exists across different grating periods. Figure 1b presents the diffraction field of a grating with a 4 μm period when illuminated by 632 nm monochromatic light, as calculated using the FDTD method. The Talbot distance for this configuration is approximately 50 μm. Figure 1c depicts the diffraction field of a grating with a 100 μm period under identical monochromatic illumination, with the calculations performed using the angular spectrum method [22]. The Talbot distance in this case is approximately 32 mm. It is evident that the propagation patterns of the diffraction fields for both grating periods are fundamentally similar. The red regions, which are the Talbot regions, are responsible for the periodic production of self-images, while the orange regions, identified as the missing-order Talbot regions, are where stripe-like images are generated due to the absence of certain diffraction orders. Thus, the missing-order regions serve as an excellent means for displacement detection due to their substantial positional changes with respect to variations in *z*.

**Figure 2:**
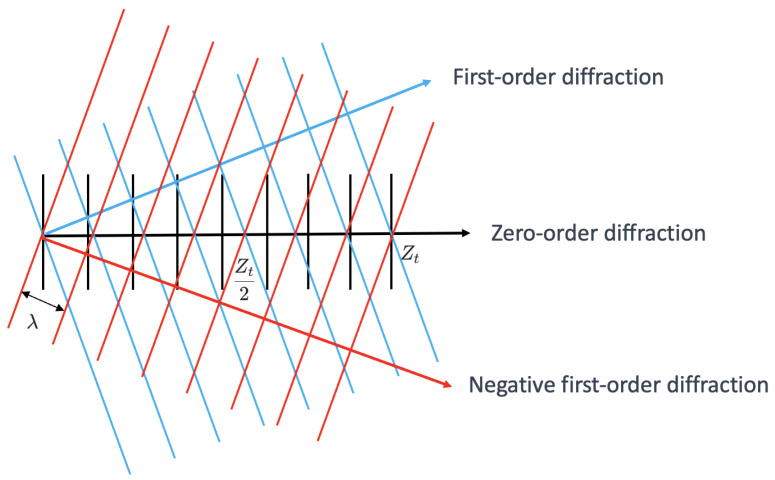
The diagram of diffraction propagation.

**Figure 3:**
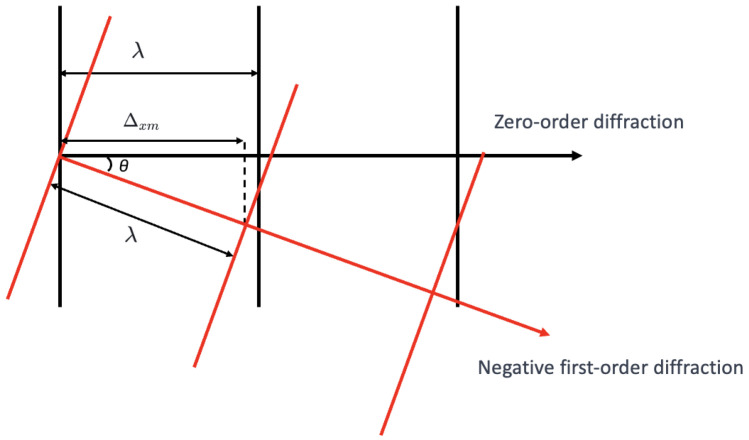
Localized amplification diagram of diffraction propagation.

Considering only the 0th- and ±1st-order diffraction waves, the diffraction propagation is shown in Figure 2. The black lines represent the 0th-order diffraction wave, the blue lines represent the +1st-order diffraction wave, and the red lines represent the −1st-order diffraction wave. The wavelength is λ, and the three waves interfere to form the Talbot positive image at the Talbot distance $Z_{t}$ and the Talbot negative image at $\frac{Z_{t}}{2}$. Figure 3 provides a detailed view of Figure 2, focusing on the explanation of the 0th- and −1st-order diffraction waves, where the angle of the −1st-order diffraction is denoted as θ.

According to the geometric relationship, the projection of the −1st-order diffracted wave on the x-axis is given by

(1) $$\begin{array}{c} \Delta_{xm}=\lambda \cos\theta \end{array}$$

where it can be derived from the grating equation that

(2) $$\begin{array}{c} \sin\theta =\frac{\lambda }{d} \end{array}$$

where θ is the diffraction angle, λ is the wavelength of the light, and *d* is the grating period. Substituting the equation above, we have

(3) $$\begin{array}{c} \Delta_{xm}=\lambda \sqrt{1-\frac{\lambda^{2}}{d^{2}}} \end{array}$$

The phase difference between the −1st-order diffracted wave and the 0th-order diffracted wave is

(4) $$\begin{array}{c} \Delta_{\phi }=\lambda -\Delta_{xm}=\lambda \left(1-\sqrt{1-\frac{\lambda^{2}}{d^{2}}}\right) \end{array}$$

The phase difference between the +1st-order diffracted wave and the 0th-order diffracted wave is the same as the equation above.

When the three groups of diffracted waves interfere, under certain conditions, where the propagation distance satisfies nλ, as the least common multiple of $\Delta_{\phi }$, they have the same phase and superimpose to form bright fringes [23]. At this distance, $Z_{t}$, it satisfies

(5) $$\begin{array}{c} Z_{t}=\frac{\lambda^{2}}{\Delta_{\phi }}=\frac{\lambda }{1-\sqrt{1-\frac{\lambda^{2}}{d^{2}}}}\approx \frac{2d^{2}}{\lambda } \end{array}$$

This distance is known as the Talbot distance, where the grating exhibits positive images at a distance of $nZ_{t}$ and negative images at a distance of $\left(n-\frac{1}{2}\right)Z_{t}$.

**Figure 4:**
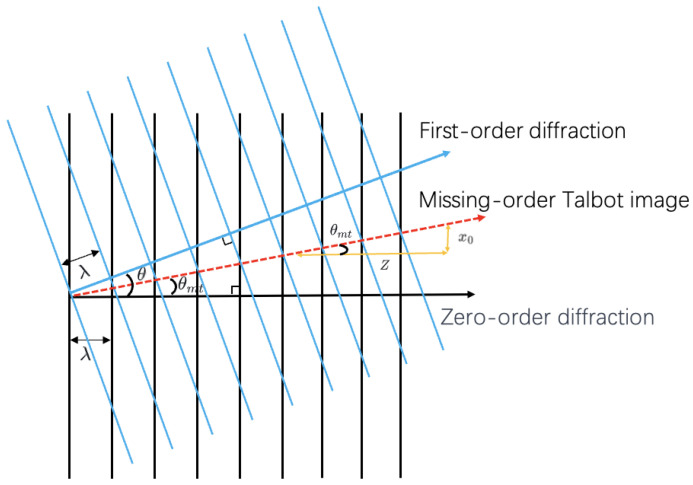
The red dashed lines depict the stripe-like patterns that arise from the interference between the 0th and +1st orders, characterizing the missing-order Talbot images.

In the region of missing-order Talbot, when only the 0th-order and +1st-order (−1st-order) diffracted waves interfere, as shown in Figure 4, the black line represents the 0th-order diffracted wave and the blue line represents the +1st-order diffracted wave, both with a wavelength of λ. The +1st-order diffraction angle is denoted as θ, and the red dashed line represents the stripe-like pattern formed by their interference. The angle of the stripe pattern, denoted as $\theta_{mt}$, can be derived from the triangle congruence theorem, and it is given by $\theta_{mt}=\frac{\theta }{2}$. Along the direction of the dashed line, which is the bisector of the propagation angles of the two diffracted waves, there will be phase overlap within the wavelength scale, resulting in the generation of the stripe-like pattern, known as the missing-order Talbot effect.

### 2.2. Principles of Displacement Detection and Displacement Extraction Algorithm

The displacement in distance *Z* results in a lateral offset of the missing-order Talbot image $x_{0}$, also shown in Figure 4. Based on the trigonometric relationships, we can derive the following equation:

(6) $$x_{0}=Z\tan\left(\frac{\theta }{2}\right)$$

Based on the grating equation, it is known that

(7) $$sin\left(\theta \right)=\frac{\lambda }{d}$$

According to the properties of frequency-domain transformations, a spatial shift is equivalent to a change in the phase of the frequency domain, and the shift *x* can be extracted from the phase in the frequency domain. Let the spatial domain image be f(x), and after a shift of $x_{0}$, it is represented as $f(x+x_{0})$. The corresponding relationship after undergoing a fast Fourier transform FFT is as follows:

(8) $$f\left(x\right)\overset{F}{⟷}F\left(\omega \right)$$

(9) $$f\left(x+x_{0}\right)\overset{F}{⟷}F\left(\omega \right)\cdot e^{j\omega x_{0}}$$

where $\omega =\frac{2\pi n}{N}$, *N* is the width of the image in pixels, and *n* is the pixel position. Let the phase slope be represented as $k_{\phi }$, and by substituting, we can find that

(10) $$k_{\phi }=angle\left(e^{j\omega x_{0}}\right)=\frac{2\pi x_{0}}{N}$$

Substituting into the above equation gives us

(11) $$x_{0}=\frac{k_{\phi }N}{2\pi }$$

Combining the above equation, we can derive that

(12) $$Z=\frac{k_{\phi }N}{2\pi }tan^{-1}\left[\frac{arcsin(\frac{\lambda }{d})}{2}\right]$$

Our algorithm, as described in Algorithm 1, integrates summation and frequency domain-processing operations to enhance the efficiency and noise resistance of the displacement measurement process. The rationale behind the summation step is that the variation information is predominantly aligned perpendicular to the grating direction. By concentrating on this specific direction, we can significantly improve the algorithmic efficiency while also leveraging the inherent noise reduction capabilities of the frequency domain. The issue of 2π ambiguity in the phase slope is related to the sampling frequency, and the introduction of frequency-domain filtering can significantly mitigate this effect.
**Algorithm 1** Missing-order Talbot Displacement Extraction Algorithm1:Obtained missing-order regions $I_{0}$ and $I_{1}$ from the displacement images.2:Summing the images $I_{0}$ and $I_{1}$ perpendicular to the direction of change yields $S_{0}$ and $S_{1}$, respectively.3:Applying Fourier transform to $S_{0}$ and $S_{1}$ results in $FT_{0}$ and $FT_{1}$, respectively.4:Calculate the frequency domain phase information by computing $\phi =\mathrm{angle}(FT_{0}/FT_{1})$5:Compute the frequency domain phase slopes $k_{\phi }=\phi \cdot FT_{0}$6:Calculate the displacement $Z=\frac{k_{\phi }N}{2\pi }tan^{-1}\left[\frac{arcsin(\frac{\lambda }{d})}{2}\right]$

## 3. Simulation and Performance Analysis

**Figure 5:**
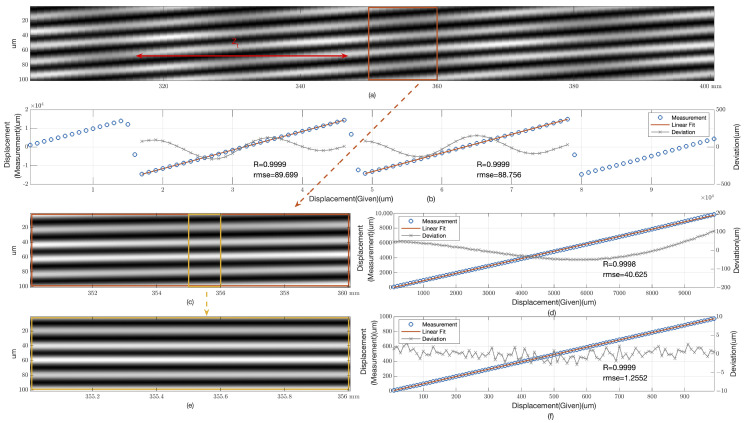
Simulation data of diffraction fields and detection results for a grating with a period of 100 μm under 632 nm plane wave illumination. (**a**) Shows the diffraction field in the missing-order region at distances ranging from 300 mm to 400 mm from the grating, with red arrows indicating the Talbot distances. (**b**) Displays the detection results and linear fit residual analysis within a dynamic range of 300 mm to 400 mm. (**c**) Illustrates the diffraction field in the missing-order region at distances between 350 mm and 360 mm from the grating. (**d**) Presents the detection results and linear fit residual analysis within a dynamic range of 350 mm to 360 mm. (**e**) Depicts the diffraction field in the missing-order region at distances from 355 mm to 356 mm. (**f**) Shows the detection results and linear fit residual analysis within a dynamic range of 355 mm to 356 mm. The correlation coefficient *R* and root mean square error (RMSE) are provided for each dynamic range.

We employed the angular spectrum method [22] to simulate three sets of data. Each set featured a grating with a period of 100 μm and a width of 5 mm, illuminated by a light source with a wavelength of 632 nm. Gaussian noise was added to each set at a signal-to-noise ratio (SNR) of 30 dB, which is a level that closely aligns with practical scenarios, and the camera used for capturing the images was set to a pixel size of 3.45 μm. This setup ensured that the simulation closely mirrored real-world conditions, allowing for the assessment of the algorithm’s performance under typical environmental noise levels. The first set included simulations over distances ranging from 300 mm to 400 mm, with an image interval of 1 mm. The second set covered a range from 350 mm to 360 mm, with an interval of 100 μm. The third set focused on a narrow range from 355 mm to 356 mm, with an interval of 10 μm. The computational results of these simulations are presented in Figure 5.

### 3.1. Accuracy and Dynamic Range Analysis

Figure 5a captures the diffraction fields within the missing-order region for the initial dataset. By focusing on a 500 μm wide section of the data, we were able to compute the displacement with high precision. Figure 5b illustrates the computational results, highlighting the periodic nature of the displacement measurements. The correlation coefficient *R* of 0.9999 indicates a very strong linear relationship between the given displacement and the measured values. The root mean square error (RMSE) of approximately 89 μm within a 30 mm dynamic range serves as an indication of the method’s accuracy, particularly given that this range is near the Talbot distance $Z_{t}$, which is a pivotal parameter in measurements based on the Talbot effect. The distinct cosine distribution of the residuals suggests a consistent and predictable pattern, which is valuable for further refinement of the measurement technique.

Figure 5c,d showcase the results for the second dataset, where the propagation angles are clearly visible. The high correlation coefficient *R* of 0.9998 and an RMSE of approximately 40 μm over a 10 mm dynamic range further validate the method’s reliability. The fluctuating pattern of the residuals indicates the method’s consistent performance across a broader range of measurements.

Figure 5e,f present the findings for the third dataset, which exhibit near-linear propagation. The correlation coefficient *R* of 0.9999 within a 1 mm dynamic range, along with an RMSE of merely 1.25 μm, underscores the method’s exceptional precision and potential for high-precision applications.

**Figure 6:**
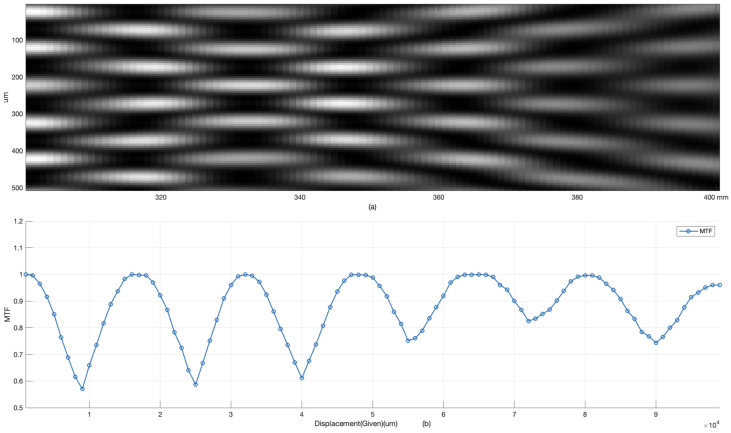
Talbot zone diffraction and MTF analysis. (**a**) Talbot zone diffraction exhibiting periodic self-imaging. (**b**) MTF representation of periodic intensity fluctuations across displacement.

We present a comparative analysis between the missing-order Talbot effect and traditional Talbot zone detection methods, highlighting the superior advantages of our approach. Figure 6a showcases the diffraction patterns within the traditional Talbot zone, where periodic self-imaging is observed. The contrast within this zone is periodically modulated and is quantified using the Modified Transfer Function (MTF), calculated as $MTF=\frac{MaxP-MinP}{MaxP+MinP}$, with MaxP indicating the peak intensity and MinP indicating the trough intensity. Figure 6b illustrates the MTF values as a function of displacement, revealing a periodic fluctuation. This pattern, though theoretically capable of displacement measurement, is outperformed by the missing-order detection method in terms of linearity and dynamic range. Our approach, leveraging the missing-order Talbot effect, excels by offering enhanced linearity and a broader dynamic range, making it a more robust solution for displacement measurement applications.

These findings collectively demonstrate the robustness of our method in displacement measurement across different scales, emphasizing its high precision and reliability. The method’s capacity to sustain high correlation coefficients and minimal root mean square error (RMSE) across diverse dynamic ranges marks a substantial advancement in the domain of optical displacement measurement, particularly within the context of Talbot effect-based metrology. The distinct cosine distribution of residuals across different datasets also opens avenues for further research to potentially enhance the method’s accuracy.

### 3.2. Algorithm Efficiency Analysis

**Figure 7:**
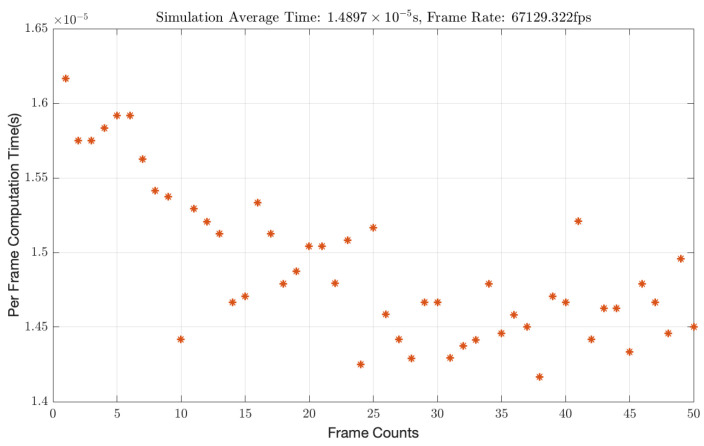
Algorithmic efficiency of displacement extraction algorithm.

In the realm of displacement measurement, temporal resolution is of critical importance, as higher resolution enables the detection of displacement information at higher frequencies. This section provides an analysis of the extraction speed and computational efficiency of our proposed algorithm. Characterized by its requirement for just a single fast Fourier transform (FFT), our algorithm demonstrates minimal computational complexity, thereby achieving a high level of computational efficiency. The accompanying Figure 7 delineates the extraction duration for a dataset comprising 50 frames, with the per-frame extraction time surpassing the threshold of 0.000015 s. This efficiency facilitates the extraction of over 67,000 frames per second, thereby enabling the capability to measure displacements at exceedingly high frequencies. The computational analysis was carried out with MATLAB R2022a, leveraging its robust set of mathematical and computational tools, all on a system driven by the Apple M2 chip.

## 4. Experimental Preparation and Analysis

**Figure 8:**
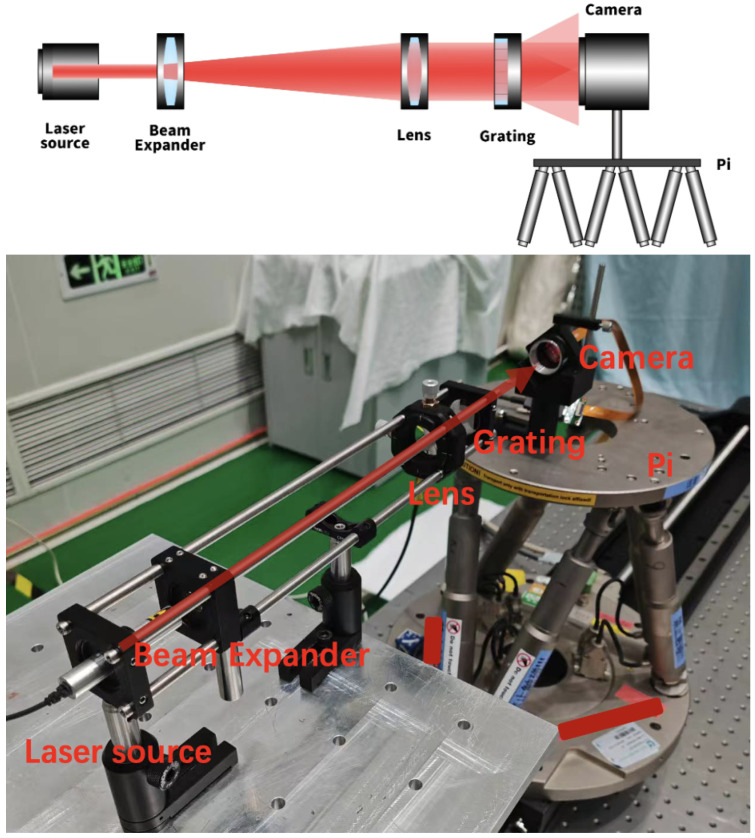
Physical experimental setup for detecting relative displacement changes between grating and camera.

The experimental optical setup, as illustrated in Figure 8, is designed to meticulously capture the nuances of the missing-order Talbot effect for displacement measurements. A 632 nm laser diode module (Thorlabs CPS635R, Newton, MA, USA) is utilized within the setup as a stable light source, offering an output power of 1.2 mW. The light beam initially undergoes expansion by a beam expander (Thorlabs LA1131-B, Newton, MA, USA), transforming it into a spherical wavefront. Subsequently, a precision collimating lens (Thorlabs LA1608-B, Newton, MA, USA) is employed to convert this spherical wavefront into a plane wavefront, ensuring the consistency of the beam’s diameter at various distances from the collimating lens, thereby guaranteeing the collimation of the light source.

The collimated beam is directed towards a meticulously crafted grating, characterized by a period of 100 μm and a width of 5 mm. This grating plays a pivotal role in producing the diffraction pattern associated with the missing-order Talbot effect. The pattern is then captured by a high-resolution camera, the Foctek FTBA20MU102, which, with pixels as small as 3.45 μm, is well suited to the task of capturing detailed imaging capabilities, crucial for the analysis of the diffraction patterns.

The camera is securely mounted on a six-degree-of-freedom platform (Pi H840), which is pivotal for the experimental process. This platform, known for its precision, allows the camera to be positioned with an accuracy of 0.1 μm, a level of control that is paramount for capturing the minute changes in the diffraction pattern that indicate displacement.

The entire setup is meticulously aligned to ensure that the collimated beam interacts optimally with the grating, and the camera is positioned to capture the resulting diffraction field. This precise collimation and alignment are critical to avoid introducing displacement measurement errors that could arise from beam propagation at oblique angles.

This system allowed for the precise collection of two distinct sets of data: the first set with a displacement interval of 100 μm over a span of 1 mm, capturing 10 frames; and the second set with a 1 mm interval over a span of 10 mm, also capturing 10 frames.

**Figure 9:**
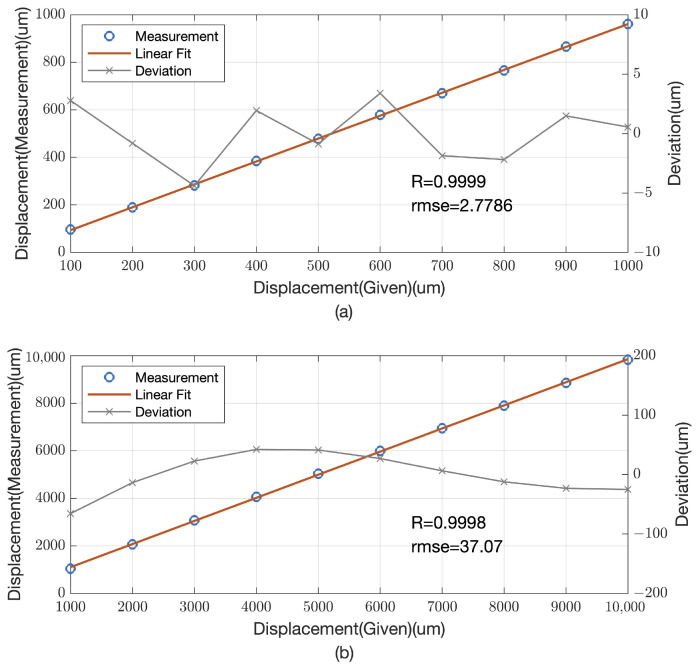
Measurement results of different dynamic ranges. (**a**) Measurement results of 1 mm dynamic range. (**b**) Measurement results of 10 mm dynamic range.

Figure 9a,b present the computational outcomes of these measurements. In the case of a 1 mm dynamic range, as shown in Figure 9a, our method achieved an impressive correlation coefficient *R* of 0.9999, with a root mean square error (RMSE) of 2.7786 μm. This slight increase in RMSE compared to simulated results could be due to environmental factors such as noise and vibrations that can subtly affect the precision of the measurements.

For a broader dynamic range of 10 mm, illustrated in Figure 9b, the method maintained a high correlation coefficient *R* of 0.9998, accompanied by a more significant RMSE of 37.07 μm. Despite the increase in RMSE, the results are in harmony with our simulations, suggesting that our method is reliable and consistent over a wider range of displacements.

These results not only highlight the system’s high precision in displacement measurement but also demonstrate its robustness against potential sources of error. The consistency of the correlation coefficient *R* being close to 1 in both dynamic ranges indicates the method’s potential for accurate and reliable displacement detection, which is critical for applications in precision optics and related fields.

## 5. Discussion

In the current landscape of commercial distance measurement technology, there are several well-established devices that dominate the market. These include time-of-flight (ToF) laser ranging devices, laser trackers, and interferometers based on laser interferometry. Time-of-flight (ToF) devices are limited by the time resolution of circuits and struggle to achieve measurements at the micrometer level. Interferometric devices, on the other hand, can reach sub-micrometer precision. However, these devices often come with significant drawbacks, such as large size and high cost, which can be prohibitive for certain applications.

The Talbot effect-based distance detection stands out as a novel approach that has yet to be commercialized and is primarily utilized in laboratory settings. Traditional distance measurement methods are often constrained by limited linearity and dynamic range. Our method, leveraging the missing-order Talbot effect, offers superior linearity and an extended dynamic range. The compact nature of our approach requires minimal space, facilitating its integration into various systems for real-time measurement applications. Furthermore, our method presents a significant cost advantage over existing technologies. To our knowledge, interferometers are typically priced in the hundreds of thousands of yuan, whereas our method can be implemented at a cost level of just a few thousand yuan.

From Equations (6) and (7), it can be observed that as the grating period decreases, the angles of the diffraction orders increase. The precision in detecting the lateral shift of the self-image is independent of the grating period and is solely dependent on the pixel size of the detector. Consequently, as the grating period decreases, the precision of displacement detection is expected to improve. Simultaneously, due to the reduction in Talbot distance, the dynamic range of detection will also decrease. However, the detection in this paper is always compared to the initial value. In practical applications, comparison with the previous frame’s result is feasible. As long as the result falls within the dynamic range of the previous frame, a cumulative method of comparison can be employed to extend the detection capability to a larger dynamic range.

At present, our method achieves micrometer precision, but it holds great potential for future enhancements. By reducing the grating period, among other strategies, it is feasible to attain sub-micrometer precision in displacement measurements. This advancement could revolutionize the field by offering a cost-effective and high-precision displacement detection method that could be widely adopted across numerous domains. The exploration of the missing-order Talbot effect for displacement measurement represents a novel application and our research marks a significant step forward in this endeavor.

## 6. Conclusions

In conclusion, the method introduced in this paper, which leverages the missing-order Talbot effect for displacement measurement, stands out for its high-precision detection capabilities. This innovative approach enables the rapid adjustment of both the dynamic range and the precision of measurements by merely altering the grating period and the wavelength of the incident light. The oscillatory pattern observed in the residuals suggests that there is opportunity for further refinement, which could lead to a substantial enhancement in measurement accuracy. Additionally, by incorporating contrast information from the Talbot regions, the dynamic range of the method can be substantially broadened, and it holds the promise of enabling absolute distance measurement.

Looking ahead, the potential applications of this method are vast and span across various industries. In optical alignment, it could provide a precise and cost-effective solution for fine-tuning systems. For mechanical arm navigation, the method’s high precision and dynamic range could be instrumental in guiding robotic arms with accuracy. In the realm of system status monitoring, it could be employed to detect minute changes in structural integrity or to monitor the performance of machinery over time.

The future integration of this method into these fields could lead to significant advancements, offering a versatile tool for high-precision measurements that is both accessible and adaptable to a wide array of applications. As research and development continue, the potential for achieving sub-micrometer precision through reductions in grating periods and the exploration of multi-wavelength techniques could further expand the capabilities and applicability of this displacement detection method.

## Data Availability

Data are contained within the article.
